# Body Mass and Emotional Eating: Emotional Eater Questionnaire (EEQ) in the Polish Adolescents’ COVID-19 Experience (PLACE-19) Study

**DOI:** 10.3390/nu14040828

**Published:** 2022-02-16

**Authors:** Dominika Skolmowska, Dominika Głąbska, Dominika Guzek

**Affiliations:** 1 Department of Dietetics, Institute of Human Nutrition Sciences, Warsaw University of Life Sciences (SGGW-WULS), 159C Nowoursynowska Street, 02-776 Warsaw, Poland; dominika_skolmowska@sggw.edu.pl; 2 Department of Food Market and Consumer Research, Institute of Human Nutrition Sciences, Warsaw University of Life Sciences (SGGW-WULS), 159C Nowoursynowska Street, 02-776 Warsaw, Poland; dominika_guzek@sggw.edu.pl

**Keywords:** emotional eating, emotional eater, Emotional Eater Questionnaire (EEQ), body mass, body mass changes, national study, population-based study, adolescents, PLACE-19 Study

## Abstract

Stress caused by the Coronavirus Disease 2019 (COVID-19) pandemic may lead to emotional eating which may have a negative impact on the weight status. This study aimed to analyze the association between emotional eating and body mass, as well as changes in body mass during the COVID-19 pandemic, within the Polish Adolescents’ COVID-19 Experience (PLACE-19) Study. A total of 1126 Polish adolescents, aged 15–20, were included. A random quota sampling was performed within a national sample, and emotional eating was assessed using Emotional Eater Questionnaire (EEQ). Based on the declared height and weight before and during the pandemic, the respondents were categorized according to their body mass (malnourished, normal weight, overweight, obese) and changes in body mass during the COVID-19 pandemic (lost weight, no body mass change, gained weight). Higher EEQ scores were achieved by female respondents compared with males (*p* < 0.0001), respondents who stated that they gained weight during the pandemic compared with those who stated either weight loss or no body mass change (*p* < 0.0001), and overweight and obese respondents compared with those who had normal weight and who were malnourished (*p* < 0.0001). A higher share of emotional eaters and very emotional eaters was found among female respondents, respondents stating weight gain during the pandemic, and overweight and obese respondents, compared with the other ones (*p* < 0.0001). Based on the findings, it may be concluded that among Polish adolescents gender, body mass, and body mass change during the COVID-19 pandemic are the major determinants of emotional eating behaviors and that female individuals, obese individuals, and those gaining weight are especially vulnerable to emotional eating behaviors. The results of the study suggest that the issue of emotional eating should be addressed in general public health policy and appropriate education should be provided to vulnerable groups such as female and obese adolescents.

## 1. Introduction

Emotional eating is defined as the tendency to eat in response to negative emotions, while the food products chosen for eating are mostly energy-dense [1], and high in sugars and fat [2]. Emotional eating has been observed in both individuals with a normal weight and those who are overweight/obese [3], and may be considered a maladaptive coping strategy [4]. As such eating behavior involves the consumption of high-calorie food products, it may even have an impact on weight status, as indicated by in the review of Frayn & Knäuper [5] that emotional eating in adults is associated with weight gain over time. However, a systematic review based on adolescents by Limbers & Summers [6] did not confirm this relationship.

The outbreak of the Coronavirus Disease 2019 (COVID-19) pandemic has caused profound psychological distress worldwide [7]. It has been shown that stress may induce changes in eating habits and may lead to either an increase or a decrease in food intake, as it was proven in the case of children and adolescents by the systematic review and meta-analysis by Hill et al. [8]. Furthermore, psychological stress, resulting from the COVID-19 pandemic and associated with anxiety, fear, and isolation [9], has been found to increase the risk of obesity, due to its influence on not only eating behaviors but also on the immune and endocrine system [10]. 

Emotional eating is mainly assessed using dedicated questionnaires, including Emotional Eating Scale (EES) [11], Salzburg Emotional Eating Scale (SEES) [12], or Emotional Overeating Questionnaire (EOQ) [13]. However, one of the most commonly used psychological tools for the assessment of emotional eating is the Emotional Eater Questionnaire (EEQ) developed by Garaulet et al. [14]. EEQ has already been recognized as a reliable tool and it has been used by various researchers during the period of the COVID-19 pandemic [15,16]. For instance, Özcan & Yeşİlkaya [15], who conducted a study on Turkish adults, indicated that during the COVID-19 pandemic a higher share of participants adopted emotional eating behaviors, compared to the prepandemic period. In the study of López-Moreno et al. [16], carried out on Spanish adults, during the COVID-19 confinement, 21.8% and 11% of the study participants were claimed as emotional eaters or very emotional eaters, respectively. 

As stated by the United Nations Children’s Fund (UNICEF), the COVID-19 pandemic has brought along several problems, such as social isolation, uncertainty, fear, and increased screen time, all of which affect the mental health of children and adolescents [17]. It is also indicated that the pandemic may continue to have long-term adverse consequences for children and adolescents when compared to adults [18]. Additionally, although some studies have assessed emotional eating behaviors in adults during the period of COVID-19 pandemic [15,16], to our best knowledge, the results of analogous studies on children and adolescents are scarce [19]. Moreover, as adolescence is a critical stage in terms of the development of emotional eating, it would be particularly important to investigate the influence of emotional eating on weight status among adolescents [20,21]. 

Taking the above into account, this study aimed to analyze the association between emotional eating and body mass, as well as changes in body mass during the COVID-19 pandemic, in a population-based sample of Polish adolescents, within the Polish Adolescents’ COVID-19 Experience (PLACE-19) Study. 

## 2. Materials and Methods

### 2.1. Ethical Statement

The study was conducted at the Institute of Human Nutrition Sciences, Warsaw University of Life Sciences (WULS-SGGW). The study was carried out in accordance with the guidelines laid down in the Declaration of Helsinki and all procedures involving human subjects received the approval of the Ethics Committee of the Central Clinical Hospital of the Ministry of Interior and Administration in Warsaw (No. 2/2021). Both participants and their parents or legal guardians provided written informed consent before participating in the study.

### 2.2. Study Population

The PLACE-19 Study, which analyzed the various aspects of the life of Polish adolescents (aged 15–20, a typical age for education in the secondary school) in the period of the COVID-19 pandemic, was divided into three phases. During the first phase of the study, the hygienic and personal protective behaviors of adolescents were investigated [22,23,24], while in the second and third phase their eating habits [25,26,27,28,29,30] and psychological aspects of eating behaviors were analyzed, respectively. 

The study of psychological aspects of eating behaviors was carried out from 21 January 2021 to 17 February 2021. Sampling was based on a random quota sampling method with quotas set for voivodeships and counties. The recruitment process included two stages: in the first stage, five counties were randomly selected (out of each 16 Polish voivodeships, resulting in a total of 80 counties), and in the second stage, five secondary schools were randomly selected (out of each randomly selected county, resulting in a total of 400 secondary schools). 

The Headmaster of each selected secondary school was sent an invitation for the school to take part in the study. If the school was willing to participate, a link to the electronic version of the survey was sent. 

The inclusion criteria for the study were as follows: female and male students of the randomly chosen secondary schools; aged 15–20 years; and informed consent obtained from both students and their parents/legal guardians.

The exclusion criteria were as follows: any missing or unreliable data in the questionnaire and participation in any previous phase of the PLACE-19 Study. 

**Figure 1:**
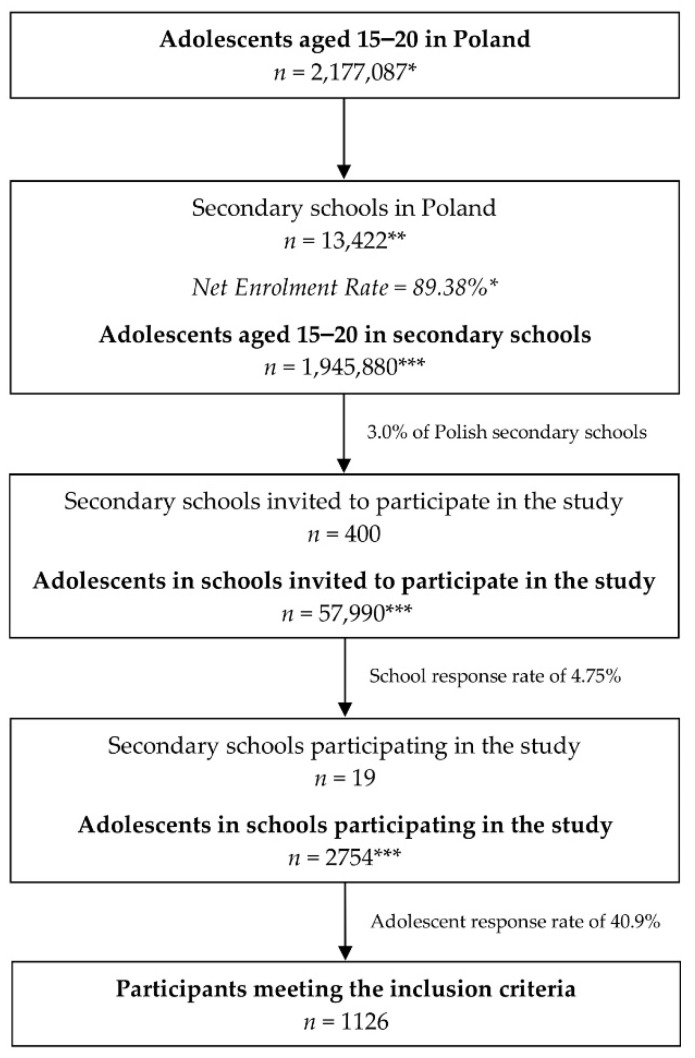
The detailed procedure of sampling of secondary schools and students within schools in the PLACE-19 Study; * data by the Central Statistical Office (CSO) in Poland [33,34]; ** data by the Polish Ministry of National Education [35]; *** calculated based on data by the CSO.

The procedure of sampling of secondary schools and students within the schools is described in detail in Figure 1. In the conducted study, a total number of 1126 adolescents participated, including 818 female and 308 male individuals. A higher proportion of girls than boys in nutritional studies is typical for Poland [31], and it was observed also in the other studies during the COVID-19 pandemic [32]. The mean age in the studied group was 16.7 ± 1.13 years (median of 17.0, differing from 15.0 to 20.0; nonparametric distribution), and it did not differ between female (16.7 ± 1.14 years, median of 17.0, differing from 15.0 to 20.0; nonparametric distribution) and male individuals (16.7 ± 1.10 years, median of 16.5, differing from 15.0 to 20.0; nonparametric distribution) (Mann-Whitney U test, *p* = 0.4110).

In the online survey, each participant was asked about his/her body mass (in kg) and height (in cm) for the period of COVID-19 pandemic (moment when the study was conducted, i.e., February 2021) as well as for the prepandemic period (March 2020 in Poland). For each respondent, for both periods the body mass index (BMI) values were calculated, using the Quetelet equation (body mass (kg)/height^2^ (m^2^)) [36]. In the case of minor adolescents (aged <18 years), the BMI was analyzed based on the gender- and age-specific Polish growth reference values [37] using OLAF software (Children’s Memorial Health Institute, Warsaw, Poland) [38] in order to specify the BMI percentile. In case of adolescents aged >18 years, the BMI values were directly analyzed. Based on the comparison of BMI percentiles (for adolescents aged <18 years) or BMI values (for adolescents aged >18 years) for the period of COVID-19 pandemic and for the prepandemic period, each adolescent was defined as gaining weight, loosing weight or maintaining stable weight during the COVID-19 pandemic.

Separately, each participant was categorized into the following groups: malnutrition, normal weight, overweight, and obesity. In the case of minor adolescents, the BMI was interpreted based on the World Health Organization (WHO, Geneva, Switzerland) growth reference cutoffs [39] set for children and adolescents, as follows: malnutrition—BMI < 5th percentile; normal weight—BMI Є < 5th–85th percentile); overweight—BMI Є < 85th–95th percentile); and obesity—BMI ≥ 95th percentile [40]. For other adolescents, the standard cutoffs fixed by WHO were applied [41].

### 2.3. Applied Questionnaire

When the PLACE-19 Study was conducted, education in all secondary schools was suspended and remote learning was introduced, as per the decision of the Polish Ministry of Education [42]. Therefore, all the data were collected using the computer-assisted web interview (CAWI) method. Students included in the study were provided with a link to the electronic version of the survey. 

Emotional eating was assessed based on EEQ, developed and validated by Garaulet et al. [14]. EEQ is used to determine the extent to which eating behaviors are affected by emotions. The questionnaire consists of 10 questions:

− Do the weight scales have a great power over you? Can they change your mood?
− Do you crave specific foods?
− Is it difficult for you to stop eating sweet things, especially chocolate?
− Do you have problems controlling the amount of certain types of food you eat?
− Do you eat when you are stressed, angry or bored?
− Do you eat more of your favourite food and with less control when you are alone?
− Do you feel guilty when eat “forbidden” foods, like sweets or snacks?
− Do you feel less control over your diet when you are tired after work at night?
− When you overeat while on a diet, do you give up and start eating without control, particularly food that you think is fattening?
− How often do you feel that food controls you, rather than you controlling food?

Each question has four possible answers, namely: (1) never, (2) sometimes, (3) generally, and (4) always, which are scored from 1 to 3 points. The maximum points that can be obtained is 30, and the higher the points, the healthier are the eating behaviors. Based on the obtained points, four groups can be distinguished, according to the original version of the questionnaire: 0–5: non-emotional eater; 6–10: low emotional eater; 11–20: emotional eater; and 21–30: very emotional eater [14]. 

### 2.4. Statistical Analysis

The respondents were stratified based on their emotional eating, as well as based on the other determinants, in the following sub-groups:

− emotional eating—non-emotional eaters (*n =* 303), low emotional eaters (*n =* 386), emotional eaters (*n =* 377), very emotional eaters (*n =* 60);
− gender—female respondents (*n* = 818) and male respondents (*n* = 308);
− declared body mass change during the COVID-19 pandemic—gained weight (*n* = 311), lost weight (*n* = 296) and no body mass change (*n* = 519);
− body mass—malnutrition (*n* = 39), normal weight (*n* = 842), overweight (*n* = 158), obesity (*n* = 87).

The normality of distribution was determined using Kolmogorov-Smirnov test. The comparison of the subgroups was performed using Mann-Whitney U test/ Kruskal-Wallis test (due to non-parametric distributions) and chi^2^ test.

The additional analysis for the detailed questions from EEQ was conducted while using multinomial logistic regression.

The statistical significance was set for the level of *p* ≤ 0.05. The statistical analysis was done using Statistica version 13.3 (StatSoft Inc., Tulsa, OK, USA), Statgraphics Plus for Windows 5.1 (Statgraphics Technologies Inc., The Plains, VA, USA) and Jamovi 2.2.5 (https://www.jamovi.org/; accessed on 6 February 2022).

## 3. Results

The emotional eating scores calculated based on the EEQ and resultant categories in the PLACE-19 Study sample, depending on gender, on declared body mass change during the COVID-19 pandemic and on body mass are presented in Table 1. Female respondents, compared with male ones, were characterized by higher EEQ scores (*p* < 0.0001), indicating that they are more vulnerable to emotional eating behaviors than male ones. At the same time, respondents who stated gain weight during pandemic, compared with those who stated either weight loss or no body mass change, received higher EEQ scores (*p* < 0.0001) and obese respondents, compared with the other ones, received higher EEQ scores (*p* < 0.0001), indicating that a higher body mass and increase of body mass are commonly associated with emotional eating behaviors.

Within the sub-groups stratified based on gender, higher share of female respondents was classified as emotional eaters and very emotional eaters, while in male sub-group as non-emotional eaters and low emotional eaters (*p* < 0.0001). Within the sub-groups stratified based on body mass change, higher share of respondents who gained weight was classified as emotional eaters and very emotional eaters, while higher share of respondents who lost weight or observed no body mass change were classified as non-emotional eaters and low emotional eaters (*p* < 0.0001). Within the sub-groups stratified based on body mass, higher share of overweight and obese respondents, compared with the other ones, were classified as emotional eaters and very emotional eaters, while higher share of malnourished and normal weight respondents were classified as non-emotional eaters and low emotional eaters (*p* < 0.0001).

As all the studied factors were associated with EEQ, additionally, to verify if there is an association between the studied factors, the association between gender, the declared body mass change during the COVID-19 pandemic, and body mass were studied (chi^2^ test). The results of the analysis revealed that although gender was not associated with body mass (*p* = 0.4121), it was associated with body mass change (*p* = 0.0015). This indicates that gender-dependent differences and body mass change-dependent differences may be associated with each other. Similarly, the body mass was associated with body mass change (*p* = 0.0003), so the related differences of EEQ may be also associated with each other.

Taking into account observed results and the risk of interfering factors, for the detailed questions within EEQ, the estimated effects of selected predictors were assessed while using multinomial logistic regression. 

The estimated effects of selected predictors using multinomial logistic regression for the declared frequency of confirmation for EEQ question 1 ‘Weight scales having a great power over respondents and changing their mood’ in the PLACE-19 Study sample are presented in Table 2. The results of multinomial logistic regression indicated that declared frequency of confirmation of weight scales always having a great power over respondents and changing their mood was higher in female while compared with male respondents (OR 6.4050 (4.0081–10.2360); *p* < 0.001), in respondents gaining weight while compared with those reporting no body mass change (OR 2.9530 (1.8762–4.6460); *p* < 0.001), and in obese respondents while compared with normal weight ones (OR 4.8740 (2.0897–11.3690); *p* < 0.001).

The estimated effects of selected predictors using multinomial logistic regression for the declared frequency of confirmation for EEQ question 2 ‘Craving specific foods’ in the PLACE-19 Study sample are presented in Table 3. The results of multinomial logistic regression indicated that declared frequency of confirmation of always craving specific foods was higher in female while compared with male respondents (OR 2.3750 (1.2917–4.3680); *p* = 0.005).

The estimated effects of selected predictors using multinomial logistic regression for the declared frequency of confirmation for EEQ question 3 ‘Difficulty stopping eating sweet things, especially chocolate’ in the PLACE-19 Study sample are presented in Table 4. The results of multinomial logistic regression indicated that declared frequency of confirmation of always having a difficulty stopping eating sweet things, especially chocolate, was higher in female while compared with male respondents (OR 2.0720 (1.2318–3.4870); *p* = 0.006), and in respondents gaining weight while compared with those reporting no body mass change (OR 2.6020 (1.5689–4.3170); *p* < 0.001).

The estimated effects of selected predictors using multinomial logistic regression for the declared frequency of confirmation for EEQ question 4 ‘Problems controlling the amount of certain types of food they eat’ in the PLACE-19 Study sample are presented in Table 5. The results of multinomial logistic regression indicated that declared frequency of confirmation of always having problems controlling the amount of certain types of food they eat was higher in respondents gaining weight while compared with those reporting no body mass change (OR 3.3635 (1.7222–6.5690); *p* < 0.001).

The estimated effects of selected predictors using multinomial logistic regression for the declared frequency of confirmation for EEQ question 5 ‘Eating when they are stressed, angry or bored’ in the PLACE-19 Study sample are presented in Table 6. The results of multinomial logistic regression indicated that declared frequency of confirmation of always eating when they are stressed, angry or bored was higher in respondents gaining weight while compared with those reporting no body mass change (OR 1.8540 (1.0528–3.2670); *p* = 0.032), and in obese respondents while compared with normal weight ones (OR 3.1500 (1.4518–6.8350; *p* = 0.004).

The estimated effects of selected predictors using multinomial logistic regression for the declared frequency of confirmation for EEQ question 6 ‘Eating more of their favourite food and with less control when they are alone’ in the PLACE-19 Study sample are presented in Table 7. The results of multinomial logistic regression indicated that declared frequency of confirmation of always eating more of their favourite food and with less control when they are alone was higher in female while compared with male respondents (OR 3.5735 (1.9036–6.7080); *p* < 0.001), in respondents gaining weight while compared with those reporting no body mass change (OR 2.7144 (1.5990–4.6080); *p* < 0.001), and in obese respondents while compared with normal weight ones (OR 2.7163 (1.1612–6.3540); *p* < 0.021).

The estimated effects of selected predictors using multinomial logistic regression for the declared frequency of confirmation for EEQ question 7 ‘Feeling guilty when eating “forbidden” foods, like sweets or snacks’ in the PLACE-19 Study sample are presented in Table 8. The results of multinomial logistic regression indicated that declared frequency of confirmation of always feeling guilty when eating “forbidden” foods, like sweets or snacks was higher in female while compared with male respondents (OR 25.2110 (7.8750–80.7126); *p* < 0.001), in respondents gaining weight while compared with those reporting no body mass change (OR 2.2422 (1.3628–3.6890); *p* = 0.001), and in obese respondents while compared with normal weight ones (OR 2.3796 (1.1180–5.0650); *p* = 0.024).

The estimated effects of selected predictors using multinomial logistic regression for the declared frequency of confirmation for EEQ question 8 ‘Feeling less control over their diet when they are tired after work at night’ in the PLACE-19 Study sample are presented in Table 9. The results of multinomial logistic regression indicated that declared frequency of confirmation of always feeling less control over their diet when they are tired after work at night was higher in respondents gaining weight while compared with those reporting no body mass change (OR 2.6434 (1.4697–4.7540); *p* = 0.001).

The estimated effects of selected predictors using multinomial logistic regression for the declared frequency of confirmation for EEQ question 9 ‘Giving up and starting eating without control, particularly food that they think is fattening, after overeating while on a diet’ in the PLACE-19 Study sample are presented in Table 10. The results of multinomial logistic regression indicated that declared frequency of confirmation of always giving up and starting eating without control, particularly food that they think is fattening, after overeating while on a diet was higher in female while compared with male respondents (OR 3.6500 (1.6062–8.2942); *p* = 0.002), and in respondents gaining weight while compared with those reporting no body mass change (OR 4.4987 (2.3704–8.5380); *p* < 0.001).

The estimated effects of selected predictors using multinomial logistic regression for the declared frequency of confirmation for EEQ question 10 ‘Feeling that food controls them, rather than they control food’ in the PLACE-19 Study sample are presented in Table 11. The results of multinomial logistic regression indicated that declared frequency of confirmation of always feeling that food controls them, rather than they control food was higher in female while compared with male respondents (OR 3.8449 (1.4827–9.9709); *p* = 0.006), in respondents gaining weight while compared with those reporting no body mass change (OR 3.8514 (1.8006–8.2378); *p* < 0.001), and in obese respondents while compared with normal weight ones (OR 3.0463 (1.0712–8.6629); *p* = 0.037).

## 4. Discussion

Emotional eating is considered as a coping mechanism during stressful moments, and is based on some typical eating behaviors [43]. Although it has been well established that decreased appetite is commonly observed among children in relation to stress [44], the intensity of emotional eating significantly increases during the period between childhood and adolescence [45]. As perceived stress and the associated emotional eating behavior may lead to higher consumption of sugar- and fat-rich foods [46], they are identified as risk factors for the development of obesity [47]. A number of studies conducted on adults have confirmed this relationship, as emotional eating was found to be associated with an increased BMI [48,49,50]. However, no study has so far proven such dependency for children or adolescents, and the results are inconclusive [4]. The study by Nguyen-Rodriguez et al. [21] performed on American middle school students showed that no differences in emotional eating were noted between normal weight and overweight adolescents. However, the study by Braet & van Strien [51] conducted on Belgian children found that overweight and obese participants scored significantly higher for emotional eating compared to children with a normal weight. At the same time, the study by Croker et al. [52] showed that the prevalence of emotional eating was the highest among obese children, compared to underweight, normal weight, and overweight participants. Similar results were observed in the study by Geliebter & Aversa [53], in which underweight participants reported that they eat less during negative emotional states whereas overweight participants reported that they overeat while experiencing negative emotions. 

The present study showed that a higher share of emotional eaters and very emotional eaters were found among overweight and obese respondents compared to the other groups. Similarly, obese respondents more often declared a number of emotional eating behaviours. Other studies also report that emotional eating is often connected to craving for chocolates [54] and that individuals with an excessive body mass, who aim to reduce their body weight, try to avoid eating this product, as it is considered as forbidden and high in calories, while its consumption results in guilt and high dietary restrictions, as well as a negative attitude toward one’s own body [55,56]. 

Gender has been identified as a key biological determinant of emotional eating [57]. In the present study, female respondents were found to be more prone to emotional eating, compared to males. This result is in compliance with other studies [48,58,59] which have indicated that women are especially susceptible to eating in response to negative emotions. The study of Péneau et al. [48] highlighted that French adult women more likely engaged in emotional eating than men. Similarly, the study by Sze et al. [58], which was carried out among Chinese university students, revealed that women were characterized by about threefold higher likelihood of emotional eating than men. Additionally, in the study by Camilleri et al. [59], which analyzed the association between emotional eating and intake of energy-dense snacks, such association was observed to be stronger in women with depressive symptoms, compared to men with depressive symptoms. The vulnerability of women to emotional eating can be explained by changes in the level of ovarian hormones across the menstrual cycle [60] and the specific moderator effect of 5-HTTLPR genotype [61]. It should also be borne in mind that emotional eating is linked with an increased risk of eating disorders, such as binge eating [62], which is more frequent among women than men [63]. 

The findings of our study indicate that the issue of emotional eating should be addressed in high-risk groups, such as female and excessive body weight adolescents. While females may be considered as especially prone to emotional eating, excessive body mass may be treated as a consequence. Special attention should be paid to female adolescents with an excessive body mass while addressing emotional eating in general public health policy. Although some important observations were formulated in the studied group, an association was observed between gender and body mass change during the COVID-19 pandemic, which may be mentioned as a source of bias within the study and may have interfered with the results. Therefore, further studies, especially prospective ones, are needed to analyze the relationship between emotional eating and body mass change. Moreover, self-report bias must be indicated, both for body mass and emotional eating behaviors, which especially in case of body mass, may be a serious problem, as participants may underestimate or overestimate their body mass. However, it is a common problem in population-based studies, but if the aim of the study is to assess the large sample from the whole country and representative for all the regions, this bias often must be accepted, as simplified model of compromised accuracy is chosen [31].

## 5. Conclusions

Thus, it may be concluded that among Polish adolescents gender, body mass, and body mass change during the COVID-19 pandemic are the major determinants of emotional eating behaviors and that female individuals, obese individuals, and those gaining weight are particularly more vulnerable to emotional eating behaviors than others. Therefore, emotional eating should be addressed in general public health policy and appropriate education should be provided to vulnerable groups such as female and obese adolescents.

## Figures and Tables

**Table 1:** The emotional eating scores calculated based on the Emotional Eater Questionnaire (EEQ) and resultant categories in the PLACE-19 Study sample (*n* = 1126), depending on gender, on declared body mass change during the COVID-19 pandemic and on body mass assessed based on the Body Mass Index (BMI).

Characteristic	Mean ± SD	Median (25th–75th)	*p* *	Non-Emotional Eaters (*n* = 303)	Low Emotional Eaters (*n* = 386)	Emotional Eaters (*n* = 377)	Very Emotional Eaters (*n* = 60)	*p* **
Depending on gender								
Female	10.5 ± 6.1	10.0 * (6.0–14.0)	<0.0001	182 (22.2%)	276 (33.7%)	305 (37.3%)	55 (6.7%)	<0.0001
Male	7.6 ± 5.3	7.0 * (3.3–10.8)		121 (39.3%)	110 (35.7%)	72 (23.4%)	5 (1.6%)	
Depending on declared body mass change during the COVID-19 pandemic								
Lost weight	9.2 ± 5.8	9.0 * (5.0–13.0) ^b^	<0.0001	171 (32.9%)	200 (38.5%)	130 (25.0%)	18 (3.5%)	<0.0001
No body mass change	8.4 ± 5.4	8.0 * (5.0–11.0) ^b^		77 (26.0%)	109 (36.8%)	98 (33.1%)	12 (4.1%)	
Gained weight	12.1 ± 6.4	12.0 * (7.0–17.0) ^a^		55 (17.7%)	77 (24.8%)	149 (47.9%)	30 (9.6%)	
Depending on body mass assessed based on the BMI								
Malnourished	6.7 ± 4.3	6.0 (4.0–10.0) ^a^	<0.0001	15 (38.5%)	16 (41%)	8 (20.5%)	0 (0.0%)	<0.0001
Normal weight	9.3 ± 5.9	8.0 * (5.0–13.0) ^b^		237 (28.1%)	300 (35.6%)	266 (31.6%)	39 (4.6%)	
Overweight	10.5 ± 6.5	10.0 * (5.0–14.0) ^b^		40 (25.3%)	49 (31.0%)	54 (34.2%)	15 (9.5%)	
Obese	12.9 ± 5.9	12.0 (9.0–18.0) ^c^		11 (12.6%)	21 (24.1%)	49 (56.3%)	6 (6.9%)	

* Mann-Whitney U test/ Kruskal-Wallis test; values with different letters (^a, b, c^) are significantly different; ** chi^2^ test.

**Table 2:** Estimated effects of selected predictors using multinomial logistic regression for the declared frequency of confirmation for Emotional Eater Questionnaire (EEQ) question 1 ‘Weight scales having a great power over respondents and changing their mood’ in the PLACE-19 Study sample (*n* = 1126).

Variable	Categories	Declared ‘Sometimes’ OR (*p*-Value)	95% Confidence Interval		Declared ‘Generally’ OR (*p*-Value)	95% Confidence Interval		Declared ‘Always’ OR (*p*-Value)	95% Confidence Interval	
			Lower Bound	Upper Bound		Lower Bound	Upper Bound		Lower Bound	Upper Bound
Gender	Male (ref.)	-	-	-	-	-	-	-	-	-
	Female	2.2400 (<0.001)	1.6109	3.1160	4.6380 (<0.001)	3.0521	7.0460	6.4050 (<0.001)	4.0081	10.2360
Body mass change	No body mass change (ref.)	-	-	-	-	-	-	-	-	-
	Lost weight	1.4730 (0.056)	0.9907	2.1890	1.8770 (0.006)	1.1999	2.9360	2.4480 (<0.001)	1.5366	3.8990
	Gained weight	1.1470 (0.501)	0.7689	1.7120	2.2070 (<0.001)	1.4280	3.4110	2.9530 (<0.001)	1.8762	4.6460
Body mass	Normal weight (ref.)	-	-	-	-	-	-	-	-	-
	Malnourished	0.6550 (0.321)	0.2837	1.5110	0.8840 (0.789)	0.3568	2.1890	0.7050 (0.506)	0.2512	1.9770
	Overweight	1.3030 (0.270)	0.8137	2.0880	1.2330 (0.447)	0.7180	2.1190	1.7040 (0.051)	0.9982	2.9100
	Obese	2.5960 (0.024)	1.1355	5.9330	3.9700 (0.001)	1.7088	9.2250	4.8740 (<0.001)	2.0897	11.3690

OR—Odds Ratio.

**Table 3:** Estimated effects of selected predictors using multinomial logistic regression for the declared frequency of confirmation for Emotional Eater Questionnaire (EEQ) question 2 ‘Craving specific foods’ in the PLACE-19 Study sample (*n* = 1126).

Variable	Categories	Declared ‘Sometimes’ OR (*p*-Value)	95% Confidence Interval		Declared ‘Generally’ OR (*p*-Value)	95% Confidence Interval		Declared ‘Always’ OR (*p*-Value)	95% Confidence Interval	
			Lower Bound	Upper Bound		Lower Bound	Upper Bound		Lower Bound	Upper Bound
Gender	Male (ref.)	-	-	-	-	-	-	-	-	-
	Female	1.7890 (0.017)	1.1088	2.8850	2.1780 (0.002)	1.3349	3.5550	2.3750 (0.005)	1.2917	4.3680
Body mass change	No body mass change (ref.)	-	-	-	-	-	-	-	-	-
	Lost weight	0.4930 (0.008)	0.2912	0.8330	0.5830 (0.048)	0.3414	0.9960	0.7530 (0.395)	0.3918	1.4480
	Gained weight	0.9370 (0.834)	0.5082	1.7270	1.3870 (0.297)	0.7499	2.5660	1.727 (0.133)	0.8472	3.5200
Body mass	Normal weight (ref.)	-	-	-	-	-	-	-	-	-
	Malnourished	0.5330 (0.205)	0.2017	1.4100	0.4600 (0.130)	0.1683	1.2560	0.1200 (0.053)	0.0141	1.0310
	Overweight	1.0140 (0.965)	0.5370	1.9160	0.6600 (0.219)	0.3407	1.2800	0.9320 (0.858)	0.4315	2.0130
	Obese	1.4470 (0.459)	0.5443	3.8470	1.1090 (0.839)	0.4102	2.9960	1.3630 (0.587)	0.4457	4.1680

OR—Odds Ratio.

**Table 4:** Estimated effects of selected predictors using multinomial logistic regression for the declared frequency of confirmation for Emotional Eater Questionnaire (EEQ) question 3 ‘Difficulty stopping eating sweet things, especially chocolate’ in the PLACE-19 Study sample (*n* = 1126).

Variable	Categories	Declared ‘Sometimes’ OR (*p*-Value)	95% Confidence Interval		Declared ‘Generally’ OR (*p*-Value)	95% Confidence Interval		Declared ‘Always’ OR (*p*-Value)	95% Confidence Interval	
			Lower Bound	Upper Bound		Lower Bound	Upper Bound		Lower Bound	Upper Bound
Gender	Male (ref.)	-	-	-	-	-	-	-	-	-
	Female	1.4870 (0.012)	1.0914	2.0250	1.4510 (0.056)	0.9899	2.1280	2.0720 (0.006)	1.2318	3.4870
Body mass change	No body mass change (ref.)	-	-	-	-	-	-	-	-	-
	Lost weight	0.8310 (0.278)	0.5950	1.1610	0.9970 (0.988)	0.6525	1.5220	1.1290 (0.6670)	0.6489	1.9650
	Gained weight	1.0910 (0.627)	0.7683	1.5480	1.8150 (0.004)	1.2050	2.7350	2.6020 (<0.001)	1.5689	4.3170
Body mass	Normal weight (ref.)	-	-	-	-	-	-	-	-	-
	Malnourished	0.7210 (0.353)	0.3610	1.4390	0.3160 (0.067)	0.0922	1.0850	0.2160 (0.139)	0.0283	1.6430
	Overweight	0.9960 (0.986)	0.6543	1.5170	1.3000 (0.285)	0.8035	2.1030	1.5560 (0.133)	0.8738	2.7700
	Obese	1.3580 (0.285)	0.7749	2.3780	1.3990 (0.316)	0.7262	2.6930	1.8620 (0.099)	0.8890	3.8990

OR—Odds Ratio.

**Table 5:** Estimated effects of selected predictors using multinomial logistic regression for the declared frequency of confirmation for Emotional Eater Questionnaire (EEQ) question 4 ‘Problems controlling the amount of certain types of food they eat’ in the PLACE-19 Study sample (*n* = 1126).

Variable	Categories	Declared ‘Sometimes’ OR (*p*-Value)	95% Confidence Interval		Declared ‘Generally’ OR (*p*-Value)	95% Confidence Interval		Declared ‘Always’ OR (*p*-Value)	95% Confidence Interval	
			Lower Bound	Upper Bound		Lower Bound	Upper Bound		Lower Bound	Upper Bound
Gender	Male (ref.)	-	-	-	-	-	-	-	-	-
	Female	1.5585 (0.004)	1.1568	2.1000	2.1101 (0.001)	1.3342	3.3370	1.1018 (0.758)	0.5942	2.0430
Body mass change	No body mass change (ref.)	-	-	-	-	-	-	-	-	-
	Lost weight	0.7241 (0.045)	0.5279	0.9930	0.6353 (0.096)	0.3723	1.0840	1.0905 (0.821)	0.5158	2.3060
	Gained weight	1.6521 (0.003)	1.1806	2.3120	3.8277 (<0.001)	2.4659	5.9420	3.3635 (<0.001)	1.7222	6.5690
Body mass	Normal weight (ref.)	-	-	-	-	-	-	-	-	-
	Malnourished	0.6112 (0.177)	0.2990	1.2490	0.6008 (0.422)	0.1733	2.0820	<0.001 (<0.001)	<0.001	<0.001
	Overweight	0.9656 (0.864)	0.6461	1.4430	1.8217 (0.021)	1.0929	3.0370	1.9995 (0.051)	0.9983	4.0050
	Obese	2.1307 (0.009)	1.2058	3.7650	4.3035 (<0.001)	2.2301	8.3050	1.7574 (0.331)	0.5637	5.4790

OR—Odds Ratio.

**Table 6:** Estimated effects of selected predictors using multinomial logistic regression for the declared frequency of confirmation for Emotional Eater Questionnaire (EEQ) question 5 ‘Eating when they are stressed, angry or bored’ in the PLACE-19 Study sample (*n* = 1126).

Variable	Categories	Declared ‘Sometimes’ OR (*p*-Value)	95% Confidence Interval		Declared ‘Generally’ OR (*p*-Value)	95% Confidence Interval		Declared ‘Always’ OR (*p*-Value)	95% Confidence Interval	
			Lower Bound	Upper Bound		Lower Bound	Upper Bound		Lower Bound	Upper Bound
Gender	Male (ref.)	-	-	-	-	-	-	-	-	-
	Female	1.3280 (0.062)	0.9860	1.7890	2.5260 (<0.001)	1.6256	3.9260	1.5440 (0.126)	0.8857	2.6900
Body mass change	No body mass change (ref.)	-	-	-	-	-	-	-	-	-
	Lost weight	0.8500 (0.322)	0.6166	1.1720	0.7370 (0.196)	0.4637	1.1710	0.6850 (0.246)	0.3607	1.2990
	Gained weight	1.3540 (0.085)	0.9596	1.9100	2.7570 (<0.001)	1.8213	4.1730	1.8540 (0.032)	1.0528	3.2670
Body mass	Normal weight (ref.)	-	-	-	-	-	-	-	-	-
	Malnourished	0.5040 (0.069)	0.2413	1.0550	0.4670 (0.173)	0.1560	1.3950	0.2960 (0.239)	0.0389	2.2450
	Overweight	1.1740 (0.427)	0.7902	1.7450	1.0730 (0.791)	0.6363	1.8100	1.5740 (0.180)	0.8116	3.0520
	Obese	1.5250 (0.142)	0.8683	2.6770	1.7450 (0.101)	0.8970	3.3950	3.1500 (0.004)	1.4518	6.8350

OR—Odds Ratio.

**Table 7:** Estimated effects of selected predictors using multinomial logistic regression for the declared frequency of confirmation for Emotional Eater Questionnaire (EEQ) question 6 ‘Eating more of their favourite food and with less control when they are alone’ in the PLACE-19 Study sample (*n* = 1126).

Variable	Categories	Declared ‘Sometimes’ OR (*p*-Value)	95% Confidence Interval		Declared ‘Generally’ OR (*p*-Value)	95% Confidence Interval		Declared ‘Always’ OR (*p*-Value)	95% Confidence Interval	
			Lower Bound	Upper Bound		Lower Bound	Upper Bound		Lower Bound	Upper Bound
Gender	Male (ref.)	-	-	-	-	-	-	-	-	-
	Female	1.5977 (0.003)	1.1775	2.1680	2.2497 (<0.001)	1.5072	3.3580	3.5735 (<0.001)	1.9036	6.7080
Body mass change	No body mass change (ref.)	-	-	-	-	-	-	-	-	-
	Lost weight	0.8798 (0.445)	0.6336	1.2220	1.0152 (0.945)	0.6614	1.5580	0.5706 (0.097)	0.2943	1.1060
	Gained weight	1.2583 (0.207)	0.8810	1.7970	2.9825 (<0.001)	1.9861	4.4790	2.7144 (<0.001)	1.5990	4.6080
Body mass	Normal weight (ref.)	-	-	-	-	-	-	-	-	-
	Malnourished	0.9040 (0.790)	0.4299	1.9010	0.8737 (0.784)	0.3330	2.2920	1.0937 (0.891)	0.3052	3.9200
	Overweight	1.1825 (0.416)	0.7898	1.7700	1.0917 (0.729)	0.6647	1.7930	1.2711 (0.477)	0.6560	2.4630
	Obese	2.6021 (0.002)	1.4329	4.7260	2.2613 (0.019)	1.1406	4.4830	2.7163 (0.021)	1.1612	6.3540

OR—Odds Ratio.

**Table 8:** Estimated effects of selected predictors using multinomial logistic regression for the declared frequency of confirmation for Emotional Eater Questionnaire (EEQ) question 7 ‘Feeling guilty when eating “forbidden” foods, like sweets or snacks’ in the PLACE-19 Study sample (*n* = 1126).

Variable	Categories	Declared ‘Sometimes’ OR (*p*-Value)	95% Confidence Interval		Declared ‘Generally’ OR (*p*-Value)	95% Confidence Interval		Declared ‘Always’ OR (*p*-value)	95% Confidence Interval	
			Lower Bound	Upper Bound		Lower Bound	Upper Bound		Lower Bound	Upper Bound
Gender	Male (ref.)	-	-	-	-	-	-	-	-	-
	Female	1.6501 (0.001)	1.2117	2.2473	3.1465 (<0.001)	2.0285	4.8807	25.2110 (<0.001)	7.8750	80.7126
Body mass change	No body mass change (ref.)	-	-	-	-	-	-	-	-	-
	Lost weight	1.1959 (0.316)	0.8431	1.6962	1.5927 (0.030)	1.0452	2.4270	1.7169 (0.036)	1.0346	2.8494
	Gained weight	1.3417 (0.095)	0.9504	1.8940	1.6101 (0.029)	1.0506	2.4677	2.2422 (0.001)	1.3628	3.6890
Body mass	Normal weight (ref.)	-	-	-	-	-	-	-	-	-
	Malnourished	0.6230 (0.242)	0.2821	1.3759	0.6596 (0.416)	0.2420	1.7976	0.3873 (0.211)	0.0876	1.7128
	Overweight	1.1464 (0.523)	0.7541	1.7427	1.4301 (0.162)	0.8666	2.3600	1.5800 (0.119)	0.8896	2.8061
	Obese	1.8114 (0.047)	1.0077	3.2561	3.10935 (<0.001)	1.6639	5.8100	2.3796 (0.024)	1.1180	5.0650

OR—Odds Ratio.

**Table 9:** Estimated effects of selected predictors using multinomial logistic regression for the declared frequency of confirmation for Emotional Eater Questionnaire (EEQ) question 8 ‘Feeling less control over their diet when they are tired after work at night’ in the PLACE-19 Study sample (*n* = 1126).

Variable	Categories	Declared ‘Sometimes’ OR (*p*-Value)	95% Confidence Interval		Declared ‘Generally’ OR (*p*-Value)	95% Confidence Interval		Declared ‘Always’ OR (*p*-Value)	95% Confidence Interval	
			Lower Bound	Upper Bound		Lower Bound	Upper Bound		Lower Bound	Upper Bound
Gender	Male (ref.)	-	-	-	-	-	-	-	-	-
	Female	1.0994 (0.537)	0.8135	1.4860	2.0076 (0.002)	1.2940	3.1150	1.5007 (0.169)	0.8414	2.6760
Body mass change	No body mass change (ref.)	-	-	-	-	-	-	-	-	-
	Lost weight	1.0224 (0.897)	0.7325	1.4270	1.1345 (0.577)	0.7280	1.7680	1.4963 (0.193)	0.8152	2.7460
	Gained weight	1.7637 (<0.001)	1.2621	2.4650	2.4655 (<0.001)	1.6207	3.7510	2.6434 (0.001)	1.4697	4.7540
Body mass	Normal weight (ref.)	-	-	-	-	-	-	-	-	-
	Malnourished	0.4246 (0.049)	0.1812	0.9950	0.4060 (0.148)	0.1199	1.3750	0.9451 (0.929)	0.2743	3.2560
	Overweight	1.0948 (0.657)	0.7341	1.6330	1.3002 (0.298)	0.7927	2.1330	1.2863 (0.471)	0.6484	2.5520
	Obese	2.0593 (0.008)	1.2072	3.5130	1.7693 (0.096)	0.9042	3.4620	2.0452 (0.102)	0.8683	4.8170

OR—Odds Ratio.

**Table 10:** Estimated effects of selected predictors using multinomial logistic regression for the declared frequency of confirmation for Emotional Eater Questionnaire (EEQ) question 9 ‘Giving up and starting eating without control, particularly food that they think is fattening, after overeating while on a diet’ in the PLACE-19 Study sample (*n* = 1126).

Variable	Categories	Declared ‘Sometimes’ OR (*p*-Value)	95% Confidence Interval		Declared ‘Generally’ OR (*p*-Value)	95% Confidence Interval		Declared ‘Always’ OR (*p*-Value)	95% Confidence Interval	
			Lower Bound	Upper Bound		Lower Bound	Upper Bound		Lower Bound	Upper Bound
Gender	Male (ref.)	-	-	-	-	-	-	-	-	-
	Female	1.3623 (0.050)	0.9995	1.8568	2.8575 (<0.001)	1.6820	4.8545	3.6500 (0.002)	1.6062	8.2942
Body mass change	No body mass change (ref.)	-	-	-	-	-	-	-	-	-
	Lost weight	1.0920 (0.607)	0.7813	1.5262	0.7759 (0.359)	0.4513	1.3338	0.8274 (0.656)	0.3596	1.9042
	Gained weight	2.1135 (<0.001)	1.5106	2.9569	2.9017 (<0.001)	1.8397	4.5768	4.4987 (<0.001)	2.3704	8.5380
Body mass	Normal weight (ref.)	-	-	-	-	-	-	-	-	-
	Malnourished	0.5897 (0.196)	0.2646	1.3138	<0.001 (<0.001)	<0.001	<0.001	0.4955 (0.500)	0.0645	3.8074
	Overweight	1.0203 (0.923)	0.6805	1.5297	1.2986 (0.356)	0.7453	2.2627	1.3592 (0.425)	0.6399	2.8871
	Obese	2.2872 (0.002)	1.3434	3.8941	3.4171 (<0.001)	1.7869	6.5348	2.2080 (0.108)	0.8405	5.8007

OR—Odds Ratio.

**Table 11:** Estimated effects of selected predictors using multinomial logistic regression for the declared frequency of confirmation for Emotional Eater Questionnaire (EEQ) question 10 ‘Feeling that food controls them, rather than they control food’ in the PLACE-19 Study sample (*n* = 1126).

Variable	Categories	Declared ‘Sometimes’ OR (*p*-Value)	95% Confidence Interval		Declared ‘Generally’ OR (*p*-Value)	95% Confidence Interval		Declared ‘Always’ OR (*p*-Value)	95% Confidence Interval	
			Lower Bound	Upper Bound		Lower Bound	Upper Bound		Lower Bound	Upper Bound
Gender	Male (ref.)	-	-	-	-	-	-	-	-	-
	Female	1.3288 (0.080)	0.9665	1.8270	2.3278 (<0.001)	1.4257	3.8007	3.8449 (0.006)	1.4827	9.9709
Body mass change	No body mass change (ref.)	-	-	-	-	-	-	-	-	-
	Lost weight	0.9408 (0.737)	0.6593	1.3426	1.359 (0.230)	0.8238	2.2420	2.0469 (0.075)	0.9313	4.4990
	Gained weight	2.1139 (<0.001)	1.5093	2.9606	3.6273 (<0.001)	2.2997	5.7213	3.8514 (<0.001)	1.8006	8.2378
Body mass	Normal weight (ref.)	-	-	-	-	-	-	-	-	-
	Malnourished	0.313 (0.031)	0.1090	0.8989	0.4111 (0.232)	0.0956	1.7683	<0.0001 (0.973)	<0.001	<0.001
	Overweight	1.2446 (0.295)	0.8263	1.8746	1.7329 (0.040)	1.0245	2.9311	2.0413 (0.068)	0.9497	4.3874
	Obese	2.8602 (<0.001)	1.6264	5.0301	5.9320 (<0.001)	3.2078	10.9698	3.0463 (0.037)	1.0712	8.6629

OR—Odds Ratio.
